# Comment on “Challenges and the Path Forward on Malaria Elimination Intervention: A Systematic Review”

**Published:** 2020-05

**Authors:** Ahad HEYDARI, Saeed FALLAH-ALIABADI

**Affiliations:** 1. Department of Health in Emergency and Disaster, School of Public Health, Tehran University of Medical Sciences, Tehran, Iran; 2. Department of Health in Emergency and Disaster, School of Public Health, Shahid Sadoughi University of Medical Sciences, Yazd, Iran

## Dear Editor-in-Chief

We read with great interest the study by SHAHANDEH et al (1), entitled “Challenges and the Path Forward on Malaria Elimination Intervention: A Systematic Review“ recently published in your journal. They conducted a valuable systematic review on challenges of Malaria Elimination Intervention. According to PRISMA checklist (2) that authors used to write this article, it is better each systematic review study has protocol registration to prevent duplicating same research while conducting and finishing the systematic reviews. POSPERO is an example of databases that each researcher can register his systematic review (3). It is much better to present search strategy for at least one database. But in this article just search terms have been presented. Searching in gray literature such as theses, committee and research reports, organization reports, conference papers, and ongoing research may provide data not published in journals. It will decrease publication bias, increase search comprehensiveness and demonstrate a balanced picture of available evidence (4). Unfortunately, we did not see enough information about above mentioned method in this article. Moreover, authors stated that 35 articles were included after reading the full text, while in PRISMA flow diagram we saw that 57 articles are included in this study. It seems the numbers in Figure 1 should revised and corrected.
